# Comparison of the Estimated Incidence of Acute Leptospirosis in the Kilimanjaro Region of Tanzania between 2007–08 and 2012–14

**DOI:** 10.1371/journal.pntd.0005165

**Published:** 2016-12-02

**Authors:** Michael J. Maze, Holly M. Biggs, Matthew P. Rubach, Renee L. Galloway, Shama Cash-Goldwasser, Kathryn J. Allan, Jo E. B. Halliday, Julian T. Hertz, Wilbrod Saganda, Bingileki F. Lwezaula, Sarah Cleaveland, Blandina T. Mmbaga, Venance P. Maro, John A. Crump

**Affiliations:** 1 Centre for International Health, University of Otago, Dunedin, New Zealand; 2 Kilimanjaro Christian Medical Centre, Moshi, Tanzania; 3 Division of Infectious Diseases, Duke University Medical Center, Durham, North Carolina, United States of America; 4 Duke Global Health Institute, Duke University, Durham, North Carolina, United States of America; 5 Centers for Disease Control and Prevention, Bacterial Special Pathogens Branch, Atlanta, Georgia, United States of America; 6 Boyd Orr Centre for Population and Ecosystem Health, Institute of Biodiversity, Animal Health and Comparative Medicine, University of Glasgow, Glasgow, United Kingdom; 7 Mawenzi Regional Referral Hospital, Moshi, Tanzania; 8 Kilimanjaro Christian Medical University College, Moshi, Tanzania; Institut Pasteur, FRANCE

## Abstract

**Background:**

The sole report of annual leptospirosis incidence in continental Africa of 75–102 cases per 100,000 population is from a study performed in August 2007 through September 2008 in the Kilimanjaro Region of Tanzania. To evaluate the stability of this estimate over time, we estimated the incidence of acute leptospirosis in Kilimanjaro Region, northern Tanzania for the time period 2012–2014.

**Methodology and Principal Findings:**

Leptospirosis cases were identified among febrile patients at two sentinel hospitals in the Kilimanjaro Region. Leptospirosis was diagnosed by serum microscopic agglutination testing using a panel of 20 *Leptospira* serovars belonging to 17 separate serogroups. Serum was taken at enrolment and patients were asked to return 4–6 weeks later to provide convalescent serum. Confirmed cases required a 4-fold rise in titre and probable cases required a single titre of ≥800. Findings from a healthcare utilisation survey were used to estimate multipliers to adjust for cases not seen at sentinel hospitals. We identified 19 (1.7%) confirmed or probable cases among 1,115 patients who presented with a febrile illness. Of cases, the predominant reactive serogroups were Australis 8 (42.1%), Sejroe 3 (15.8%), Grippotyphosa 2 (10.5%), Icterohaemorrhagiae 2 (10.5%), Pyrogenes 2 (10.5%), Djasiman 1 (5.3%), Tarassovi 1 (5.3%). We estimated that the annual incidence of leptospirosis was 11–18 cases per 100,000 population. This was a significantly lower incidence than 2007–08 (p<0.001).

**Conclusions:**

We estimated a much lower incidence of acute leptospirosis than previously, with a notable absence of cases due to the previously predominant serogroup Mini. Our findings indicate a dynamic epidemiology of leptospirosis in this area and highlight the value of multi-year surveillance to understand leptospirosis epidemiology.

## Author Summary

Leptospirosis is an infectious disease that causes a fever. It can be severe or fatal. Understanding how many people get leptospirosis helps to determine priorities in allocating resources for disease diagnosis, treatment, and prevention. There are few data about leptospirosis incidence in sub-Saharan African countries. The only mainland estimate is from northern Tanzania for the years 2007–08. To see if leptospirosis incidence had changed since 2007–08, we measured leptospirosis incidence in the same location in 2012–2014. To do this, we systematically approached people at two hospitals in the Kilimanjaro Region and tested them for leptospirosis. We adjusted the number of identified cases of leptospirosis found at the hospitals to account for people with fever who did not come to hospital for testing and care. We also adjusted for imperfect testing methods. We found that the number of people who developed leptospirosis annually had dropped from 75–102 cases per 100,000 people during 2007–08 to 11–18 cases per 100,000 people during 2012–14. Also, the subtype of leptospirosis responsible for the most cases during 2007–08 was not present during 2012–14. The number of people developing leptospirosis was not stable, highlighting the value of measuring how commonly leptospirosis occurs over several years.

## Introduction

Leptospirosis is a major cause of illness worldwide with an estimated 1.03 million cases, 59,000 deaths, and 2.90 million disability adjusted life years lost annually [1, 2]. The burden of disease is thought to be greatest in tropical countries, although reported estimates of incidence in continental Africa are scarce [3, 4]. Accurate estimates of incidence are important for estimation of disease burden and consequently, appropriate allocation of resources for diagnosis, treatment, and prevention. Challenges in estimating incidence that may account for the scarcity of reports of incidence in Africa include lack of availability of diagnostic tests [5], low clinician awareness [6], and non-specific presentation.

Although active, population-based surveillance is an ideal method for accurately determining incidence, resource and logistic challenges often preclude its use. Multiplier methods have been used successfully to estimate the incidence of acute infectious diseases in resource-limited settings by extrapolating from hospital based data [7, 8]. Specifically, multiplier methods were used to determine the incidence of acute leptospirosis in the Kilimanjaro Region during 2007–08 [9]. Using hospital based prevalence data and multipliers from a linked health-care seeking behaviour survey [10], the annual incidence of acute leptospirosis was estimated as 75–102 cases per 100,000 [9]. This estimate of incidence based on empirical data was substantially higher than an estimate (7–38 cases per 100,000 population) for Tanzania based on a modelling approach using incorporated data from a systematic review of risk factors [2]. Leptospirosis may cause endemic disease, but is also capable of causing epidemics during flooding or other extreme weather events [11]. As such, data gathered from the same location from multiple time periods can provide insights into the dynamics of disease incidence over time, distinguish periods of endemic and epidemic transmission, and help determine more representative burden of disease estimates.

We sought to estimate the incidence of acute leptospirosis in northern Tanzania from 2012 until 2014 using a similar methodology to the previous estimate in the same region in order to describe trends over multiple year periods.

## Methods

**Fig 1:**
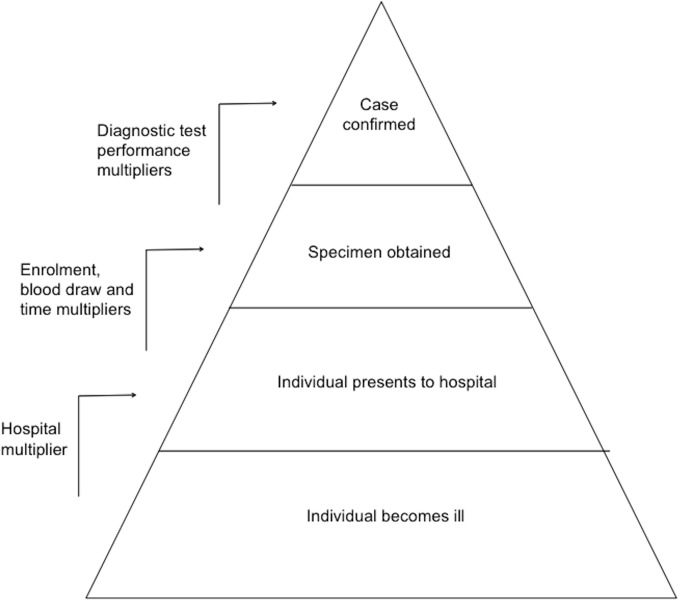
Surveillance pyramid showing multipliers used to account for incomplete case identification. Modified from Biggs HM, Hertz JT, Munishi OM, Galloway RL, Marks F, Saganda W, et al. Estimating leptospirosis incidence using hospital-based surveillance and a population-based health care utilization survey in Tanzania. PLoS Negl Trop Dis. 2013;7(12):e2589.

We calculated the incidence of acute leptospirosis using a multiplier study design. Briefly, we combined a healthcare utilisation survey performed in two districts within the Kilimanjaro Region with a hospital-based surveillance involving systematic evaluation for leptospirosis in febrile patients at the two major referral hospitals in the Kilimanjaro Region. We multiplied the number of identified cases of acute leptospirosis by a number of factors designed to account for incomplete data using the surveillance pyramid model (Fig 1.) [9].

### Fever surveillance

Study Site: We studied patients at two referral hospitals in Moshi, Tanzania. Moshi is the administrative centre for the Kilimanjaro Region that has a population of 1.6 million. Moshi is situated at an elevation of approximately 890m and has a tropical climate with rainy seasons from October through December and March through May. Aside from urban Moshi, the region is rural with inhabitants practicing cultivation and small-holder farming. Kilimanjaro Christian Medical Centre (KCMC) is a 450 bed hospital and the zonal referral centre for several regions in Northern Tanzania. Mawenzi Regional Referral Hospital (MRRH) is a 300 bed hospital and the referral centre for the Kilimanjaro region.

Enrolment procedures: From 20 February 2012 through 28 May 2014 the study team approached all adult patients who were admitted to KCMC with a febrile illness as well as all adult or paediatric patients who were admitted at MRRH. In addition we approached every second patient who presented with fever to the outpatient department at MRRH. Hospitalized participants were eligible for enrolment if they had a history of fever within the previous 72 hours or an axillary temperature of >37.5°C or a tympanic, oral or rectal temperature of ≥38.0°C at admission. Non-hospitalized patients were eligible if they had an axillary temperature of >37.5°C or a tympanic, oral or rectal temperature of ≥38.0°C. All adult study participants provided written informed consent. For those under 18 years, a parent or guardian provided written informed consent. In addition, written assent was provided for those aged 12 to 18 years. This study differed from the previous Kilimanjaro Region incidence study in its enrolment of outpatients and the enrolment of children at MRRH rather than at KCMC.

Enrolment occurred only on weekdays. Enrolled patients underwent phlebotomy, with blood allocated for acute leptospirosis serology only if there was sample available after blood parasite microscopy and blood culture. Participants were requested to return for collection of convalescent serum 4–6 weeks after enrolment. For those who did not attend the scheduled follow up, we attempted to contact them and encourage attendance. Additionally, we recorded inpatient death. Unlike the previous study estimating leptospirosis incidence in the Kilimanjaro Region, we did not record inter-hospital transfer.

Laboratory methods: Serology for leptospirosis was performed on acute and convalescent serum samples using the standard microscopic agglutination test (MAT) with a panel of 20 *Leptospira* serovars belonging to 17 serogroups at the United States Centers for Disease Control and Prevention. These included serogroups: Australis (represented by *L*. *interrogans* serovar Australis, *L*. *interrogans* serovar Bratislava), Autumnalis (*L*. *interrogans* serovar Autumnalis), Ballum (*L*. *borgpetersenii* serovar Ballum), Bataviae (*L*. *interrogans* serovar Bataviae), Canicola (*L*. *interrogans* serovar Canicola), Celledoni (*L*. *weilii* serovar Celledoni), Cynopteri (*L*. *kirschneri* serovar Cynopteri), Djasiman (*L*. *interrogans* serovar Djasiman), Grippotyphosa (*L*. *interrogans* serovar Grippotyphosa), Hebdomadis (*L*. *santarosai* serovar Borincana), Icterohaemorrhagiae (*L*. *interrogans* serovar Mankarso, *L*. *interrogans* Icterohaemorrhagiae), Javanica (*L*. *borgpetersenii* serovar Javanica), Mini (*L*. *santarosai* serovar Georgia), Pomona (*L*. *interrogans* serovar Pomona), Pyrogenes (*L*. *interrogans* serovar Pyrogenes, *L*. *santarosai* serovar Alexi), Sejroe (*L*. *interrogans* serovar Wolffi), and Tarassovi (*L*. *borgpetersenii* serovar Tarassovi).

Case definitions: We defined confirmed acute leptospirosis as participants who demonstrated a four-fold rise in agglutinating antibody titres between acute and convalescent serum samples. Cases were defined as probable if a participant’s serum had a single agglutinating titre of at least 1:800. These definitions were identical to those used to obtain the previous incidence estimate [9, 12]. The predominant reactive serogroup, for confirmed cases was defined as the serogroup containing the serovar with the largest rise in titres between acute and convalescent sera. For probable cases, we used the serovar with the highest titre to define the serogroup.

### Derivation of multipliers

A time multiplier of 1.40 was used to account for enrolment occurring only on weekdays (5 of every 7 days). Additionally a study duration multiplier of 0.44 was included to calculate annual incidence from a study that enrolled for 27 months (20 February 2012 through 28 May 2014). We applied enrolment and blood draw multipliers to account for eligible patients who either did not enrol or for whom blood was not available for leptospirosis serology. Calculations of these multipliers are presented in the results. We were unable to include a transfer multiplier in the current study as details of inter-hospital transfer of participants were not recorded. For the estimation of incidence based solely on confirmed cases, a paired sera multiplier was applied to account for those patients who did not have paired sera drawn. Diagnostic test multipliers were used to account for the sensitivity and specificity of MAT serology. The sensitivity was estimated at 100% for paired sera, 48.7% for participants with solely acute sera and 93.8% for those with solely convalescent sera. The specificity was estimated at 93.8%. The estimates are based on a published evaluation of diagnostic tests [9, 13] and matched those used in the 2007–08 study.

### Healthcare utilisation survey

A healthcare utilisation survey was carried out in the Moshi Urban (population 184,292) and Moshi Rural (population 466,737) Districts of Kilimanjaro Region between 13 June and 22 July 2011 as previously reported [9, 14]. Briefly, 30 of the 45 wards were selected randomly using a population-weighted approach. A study member collected data from the heads of the first 27 households encountered within the ward. A total of 810 households were sampled, comprising 3,919 household members. All households had at least one member >15 years of age, 361 had at least one member aged between 5 and 15 years of age, and 198 households had at least one member aged below 5 years. The demographic characteristics from the healthcare utilisation survey have been previously compared to the 2002 Tanzanian Census [9]. Age specific population data has not yet been released from the 2012 Census [14]. Questions relating to health-care seeking behaviour in the event of febrile illness were used to identify participants likely to present to KCMC or MRRH. These questions included, ‘what is the name of the health care facility with an inpatient ward where you/your family would go if you/your family had fever?’ and ‘what will you do if a [household member subdivided by age bracket] has a fever for ≧ 3 days?’. The hospital multipliers are presented in Table 1. Each multiplier is the reciprocal of the proportion of survey participants who responded that they would attend KCMC or MRH as their first or second choice healthcare provider.

**Table 1:** Multipliers based on responses to relevant questions in healthcare utilisation survey, Moshi Rural and Moshi Urban Districts, Tanzania, 2011.

Age, years	Households			KCMC	MRRH	KCMC %	MRRH %	KCMC mult.	MRRH mult.
**What will you do if a [household member] has a fever for ≧ 3 days?**									
**<5**		198	17		67	8.6	33.8	11.6	3.0
**5 to 15**		361	10		137	2.8	38.0	36.1	2.6
**≧15**		810	35		299	4.3	36.9	23.1	2.7
**What is the name of the health care facility with an inpatient ward where you/your family would go if you/your family had fever?**									
**<5**		198	10		68	5.1	34.3	19.8	2.9
**5 to 15**		361	28		133	7.8	36.8	12.9	2.7
**≧15**		810	50		313	6.2	38.6	16.2	2.6

KCMC: Kilimanjaro Christian Medical Centre

MRRH: Mawenzi Regional Referral Hospital

Mult.: multiplier

### Population denominators

We used population totals from the 2012 census [14]. As age specific population data were not available from the 2012 census, we multiplied age specific proportions from the 2002 census by the 2012 population total to estimate age-specific populations. The 2007–2008 Kilimanjaro Region incidence estimate used population totals from the 2002 census.

### Comparison of incidence between study periods

We compared incidence by using the estimate of incidence derived from confirmed and probable cases from each of the study periods and the estimated population sampled as the denominator. As shown in Table 2, the estimated population sampled was calculated by multiplying the total population by the proportion of participants in the healthcare utilization survey that identified KCMC or MRRH as hospitals they would attend in the event of febrile illness. We compared the highest estimates of incidence in each of the study periods.

**Table 2:** Estimated population of Moshi Urban and Moshi Rural District that reported that they would attend KCMC or MRRH if they developed a febrile illness during the time periods 2007–08 and 2012–14.

Age, years	Population 2007–08	Population 2012–14	Proportion attending KCMC	Proportion attending MRRH	Estimated population sampled 2007–2008	Estimated population sampled 2012–2014
<5	68,680	82,016	0.09	0.34	6,212	27,613
5 to 15	150,218	179,387	0.03	0.38	4,138	67,703
≥15	326,270	389,625	0.04	0.37	134,536	160,660
Total	545,168	651,028			144,887	255,976

KCMC: Kilimanjaro Christian Medical Centre

MRRH: Mawenzi Regional Referral Hospital

### Sensitivity Analysis

We repeated all calculations using both probable and confirmed cases and then using confirmed cases only. Additionally we performed a one-way sensitivity analysis by varying hospital multipliers according to answers to alternative relevant questions in the healthcare utilisation survey that might also reflect the behaviour of participants and diagnostic test multipliers by using a range of alternative plausible sensitivity values for MAT [15–17].

### Statistical analysis

Data was entered using the Cardiff Teleform system (Cardiff, Inc., Vista, CA, USA) into an Access database (Microsoft Corporation, Redmond, WA, USA). Incidence calculations were carried out using Microsoft Excel 2010 (Microsoft Corporation. Redmond, WA, USA) spreadsheets. Other analyses were performed using STATA, version 13.1 (STATA-Corp, College Station, TX, USA). We used a test of proportions to compare the incidence between 2007–08 and 2012–14. All p values are 2 sided and statistical significance was set at p<0.05.

### Research ethics

This study was approved by the KCMC Research Ethics Committee (#295), the Tanzania National Institutes for Medical Research National Ethics Co-ordinating Committee (NIMR1HQ/R.8cNo1. 11/283), the Institutional Review Board of Duke University Medical Center (IRB#Pro00016134) and the University of Otago Human Ethics Committee (Health) (H15/055).

## Results

### Fever surveillance

Of 1,115 participants enrolled from within the study districts, 1,017 (91.2%) had blood drawn for leptospirosis testing. Of the 1,115 participants, 409 (37.7%) <5 years, 111 (10.0%) 5–14 years, and 595 (53.4%) were aged ≥15 years. A total of 593 (46.9%) participants were male. A total of 758 (74.6%) participants reported having a fever for at least 3 days.

Of 1,017 participants tested for leptospirosis, 12 (1.2%) met the case definitions for confirmed leptospirosis and an additional 7 (0.7%) met the case definitions for probable acute leptospirosis. The predominant reactive serogroups among confirmed and probable cases of leptospirosis are summarised in Table 3.

**Table 3:** Frequency of confirmed and probable acute leptospirosis defined by predominant reactive *Leptospira* serogroup, Moshi Rural and Moshi Rural Districts, Tanzania, 2007–8 and 2012–14.

*Leptospira* serogroup	Study Year			
	2007–08 (n = 41)		2012–14 (n = 19)	
	N	(%)	N	(%)
**Mini**	15	(36.6)	0	(0)
**Australis**	14	(34.1)	8	(42.1)
**Celledoni**	4	(9.8)	0	(0)
**Autumnalis**	3	(7.3)	0	(0)
**Icterohaemorrhagiae**	1	(2.4)	2	(10.5)
**Tarassovi**	1	(2.4)	1	(5.3)
**Djasiman**	1	(2.4)	1	(5.3)
**Hebdomadis**	1	(2.4)	0	(0)
**Sejroe**	0	(0)	3	(15.8)
**Pyrogenes**	0	(0)	2	(10.5)
**Grippotyphosa**	0	(0)	2	(10.5)

Of both confirmed and probable cases, there were seven (1.7%, 95% confidence interval [CI] 0.4–2.9%) cases among 416 outpatients and 12 (1.9%, 95% CI 0.8–3.1%) cases among 601 inpatients. There was no statistically significant difference in the prevalence of leptospirosis between inpatients and outpatients (p = 0.72).

#### Multipliers

Because the residence of those who declined to participate was not recorded, the enrolment multiplier was calculated for participants residing in both study and non-study districts. As 1,420 (59.3%) of 2,394 eligible patients were enrolled, we applied an enrolment multiplier of 1.69. Age was also not recorded among those who did not participate, so age-specific enrolment multipliers were not calculated. Of the 1,115 participants enrolled, 1017 (91.2%) were tested for leptospirosis and age-specific blood draw multipliers were calculated as shown in Table 4. A paired sera multiplier of 1.60 was applied to incidence calculations involving confirmed cases only as 637 (62.6%) of 1,017 patients who had serum drawn had paired sera tested for leptospirosis. Of the 380 who were lost to follow up or were otherwise unable to provide both acute or convalescent sera, 173 (45.5%) were aged <5 years, 21 (5.5%) were aged 5–14 years, 186 (49.6%) of 380 were aged ≥15 years and 180 (47.4%) were male.

**Table 4:** Calculation of blood-draw multipliers, Moshi Rural and Moshi Urban Districts, Tanzania, 2012–14.

Age,	Enrolled	Blood Drawn	Multiplier
years	N	N (%)	
**<5**	431	350 (82)	1.2
**5–15**	111	109 (98)	1.0
**≧15**	573	558 (97)	1.0

### Incidence calculation

The annual incidence of acute leptospirosis in the Moshi Urban and Rural Districts (2012–2014) was 11–18 cases per 100,000 population using hospital multipliers derived from the question ‘To which facility would you go if you were unwell with a fever lasting ≧3 days?’. When using respnses to the question, ‘What is the name of the health care facility with an inpatient ward where you/your family would go if you/your family had fever?’ the incidence was 9–18 cases per 100,000 population. The annual incidence was highest in adults, ranging from 13 to 29 cases per 100,000 population. Details of the calculation and age-specific incidences are included in Table 5. The estimated incidence for confirmed and probable cases was (18 cases per 100,000 population) was statistically significantly lower than the estimate of 102 cases per 100,000 population from 2007–08 (p<0.0001).

**Table 5:** Calculation of incidence of acute leptospirosis, Moshi Rural and Moshi Urban Districts, Tanzania, 2012–14.

Age group, years	KCMC crude cases	KCMC adjusted cases	MRRH crude cases	MRRH adjusted cases	Total adjusted cases	Paired sera	Days/Week	Enrolment/ Blood draw	Study duration	Estimated annual cases	Population	Annual Incidence per 100,000
**Based on the question: To which facility would you go if you were unwell with a fever lasting ≥3 days?**												
**Confirmed and probable cases**												
**<5**	N/A	N/A	2	6	6	N/A	1.4	2.1	0.4	7	82,016	9
**5 to 15**	N/A	N/A	0	0	0	N/A	1.4	1.7	0.4	0	179,387	0
**≧15**	3	67	14	57	62	N/A	1.4	1.4	0.4	67	389,625	17
**Overall**										75	651,028	11
**Confirmed only**												
**<5**	N/A	N/A	1	3	3	1.6	1.4	2.1	0.4	6	82,016	7
**> = 5 to <15**	N/A	N/A	0	0	0	1.6	1.4	1.7	0.4	0	179,387	0
**≧15**	3	67	8	33	50	1.6	1.4	2.2	0.4	111	389,625	28
**Overall**										117	651,028	18
**Based on the question: What is the name of the health care facility with an inpatient ward where you/your family would go if you/your family had fever?**												
**Confirmed and Probable**												
**<5**	N/A	N/A	2	5	5	N/A	1.4	2.1	0.4	7	82,016	9
**5 to 15**	N/A	N/A	0	0	0	N/A	1.4	1.7	0.4	0	179,387	0
**≧15**	3	47	14	45	46	N/A	1.4	1.4	0.4	50	389,625	13
**Overall**	3									57	651,028	9
**Confirmed only**												
**<5**	N/A	N/A	1	2	2	1.6	1.4	2.1	0.4	7	82,016	8
**5 to 15**	N/A	N/A	0	0	0	1.6	1.4	1.7	0.4	0	179,387	0
≧15	3	47	8	40	44	1.6	1.4	2.2	0.4	113	389,625	29
**Overall**										120	651,028	18

KCMC: Kilimanjaro Christian Medical Centre

MRRH: Mawenzi Regional Referral Hospital

N/A: Not applicable

Estimates of annual incidence in six monthly time blocks is summarised in Table 6. These data show a higher incidence during the first few months of the study.

**Table 6:** Incidence of acute leptospirosis in six months intervals, Moshi Rural and Moshi Urban Districts, Tanzania, 2012–14.

Time Period	Participants	Crude Cases	Annual Incidence per 100,000
	N	N (%)	
18 Feb—30 Jun 2012*	256	8 (3.1)	24
**1 Jul– 31 Dec 2012**	222	2 (0.9)	6
**1 Jan– 30 Jun 2013**	285	4 (1.4)	8
**1 Jul– 31 Dec 2013**	218	5 (2.3)	9
**1 Jan– 30 Jun 2013**	116	0 (0)	0

* 4 month time interval

### Sensitivity Analysis

The results of the one-way sensitivity analysis are presented in Table 7. When we derived hospital multipliers from alternative questions from the healthcare utilisation survey that might also reflect the behaviour of participants, the estimated annual incidence ranged from 8–37 cases per 100,000 population. When we varied the estimated sensitivity of MAT from the lowest to highest plausible values [5, 13, 16, 18, 19], the estimated annual incidence varied from 10–25 cases per 100,000 population.

**Table 7:** Sensitivity analysis of leptospirosis incidence, Moshi Rural and Moshi Urban Districts, Tanzania, 2012–14.

Variable	Cases per 100,000 population)
**Variation in multipliers based on varying the question from the healthcare utilisation survey**	
Question: What will you do if a [household member subdivided by age bracket] has a fever lasting <3 days?	25–37
Question: What will you do if a [household member subdivided by age bracket] has a fever [duration not specified]?	32–49
**Variations in estimation of sensitivity of MAT**	
**Lowest plausible estimate**	17–25
Paired sera: 75%	
Solely acute sera: 6%	
Solely convalescent sera: 70%	
**Highest plausible estimate**	10–14
Sensitivity paired sera: 100%	
Sensitivity acute sera only: 95%	
Sensitivity convalescent sera only: 95%	

## Discussion

This study highlights the dynamic nature of leptospirosis epidemiology in the Kilimanjaro Region, Tanzania. Our study shows that both incidence and serogroup dominance among human cases have changed. The overall annual estimate of 11–18 cases of acute leptospirosis per 100,000 population is substantially lower than the estimated incidence of 75–102 cases per 100,000 population per year from 2007–08 [9]. In addition the age-specific incidence appears to have changed, with children estimated to have a lower incidence than in the 2007–08 study. Despite the lower incidence during 2012–14, leptospirosis still appears to be an important cause of fever in our region.

The explanation for the wide variation in incidence is uncertain, but may reflect changes in climatic conditions, transient presence of an infected reservoir host, changes in human-animal interactions, or changes in rodent-livestock interactions. Regarding climate, leptospirosis is recognised as an important public health problem following extreme weather events in other parts of the world such as Malaysia, Philippines, and Thailand [11]. The strong El Niño conditions of 2006–07 were associated with flooding and epidemics of other diseases, such as Rift Valley Fever. El Niño conditions may have influenced leptospirosis incidence during 2007–08 [20–22]. Urbanisation or reduced livestock ownership are unlikely to explain the change in incidence, because, firstly population increased in rural areas between the 2002 and 2012 censuses and secondly livestock numbers are thought to have increased over the study period [14, 23]. Livestock vaccination coverage to any disease remains low in Tanzania, and we think that increased vaccination against leptospirosis is unlikely to account for the reduced incidence [23].

There has also been a change in the most common predominant reactive serogroup. During 2007–08, serogroup Mini was the most commonly implicated serogroup, whereas during 2012–2014 there were no cases of leptospirosis in which Mini was the dominant serogroup [24]. Our findings are consistent with an interpretation of unstable serogroup transmission dynamics, with increased transmission of a serovar from within the Mini serogroup during 2007–08. Research to understand the reservoir hosts, sources, and risk factors for serogroup Mini in northern Tanzania may help to explain the apparent unstable transmission. Aside from the fever surveillance work at KCMC and MRRH in 2007–08, there are no reports of *Leptospira* serovars belonging to the Mini serogroup causing human disease in East Africa, although Mini serovars have been isolated from people and small mammals in the western Indian Ocean islands of Mayotte and Madagascar [25–28]. Mini is an uncommonly tested serogroup, but serological reactivity to Mini has been shown in cattle [29–32], wild game animals [33, 34], and rodents [35]. Pilot serological study of cattle slaughtered for meat in Moshi Municipal District in 2014 found seroreactivity to *L*. *borgpetersenii* serovar Mini in 14 (24.1%) out of 58 animals tested [36]. This finding indicates that livestock may be an important source for human leptospirosis in the area. In addition, our data from both humans and livestock suggests that a representative of the Mini serogroup should be included in future MAT panels in East Africa.

In 2012–14, the most common predominant serogroup was Australis, which was the second most commonly identified predominant reactive serogroup during 2007–08. Agglutination was observed against both test serovars (*L*. *interrogans* serovar Australis and *L*. *interrogans* serovar Bratislava) in 2007–08 and 2012–14. Continued identification of this serogroup suggests that at least one serovar from the Australis serogroup is endemic in the region. Serovars from the Australis serogroup have been isolated from rodents in Tanzania [37], a human in Kenya [38], an African grass rat in Nigeria [39] and cattle in Zimbabwe [40]. Serologic studies of animals in East Africa, have reported infrequent sero-reactivity to Australis serogroup in sheep, goats and pigs [41] and cattle [42]. Seroreactivity against serogroup Australis serovars was also observed in cattle slaughtered for meat in the Moshi area in 2014 [36]. Rural residence has previously been identified as a risk factor for acute leptospirosis in northern Tanzania [24]. These findings raise the possibility of livestock as an important source of infection. However, care is needed in interpreting infecting *Leptospira* strains from serological data as cross-reactions between Mini, Sejroe, and Hebdomadis serogroups are common and using serological data to infer infecting serovars is unreliable [43, 44]. Therefore, *Leptospira* spp. isolates from humans and animals, and studies investigating risk factors are needed.

One of our study’s strengths is that the estimate of incidence used the same hospital surveillance system and the same healthcare utilisation survey within the same districts as the earlier study, allowing a direct comparison of distinct time periods. Differences in enrolment practices and multipliers between the 2007–08 incidence estimate and the current study may have influenced the difference in estimated incidence. The 2012–14 study enrolled both those hospitalised and those treated as outpatients, where as the 2007–08 study enrolled only inpatients. However, in our study, the incidence did not vary by admission status. Other minor alterations in multipliers include the addition of a blood-draw multiplier and the omission of a transfer multiplier as these data were not collected. These multipliers ranged from 0.7–1.2 and if applied, would have widened the apparent difference between incidence estimates from 2007–08 and 2012–2014. The human population of the study area has been estimated from the 2012 census in the 2012–14 study and the 2002 census for the study of Biggs et al. The total population rose from 545,168 to 651,028 between 2002 and 2012 [14]. It is likely therefore that the 2007–08 study underestimated the true population at the time of the study, and consequently will have overestimated the incidence by a small margin.

There are other limitations in our study that influence interpretation of the results. We chose to estimate incidence using multiplier methods as resource limitations precluded active surveillance in the entire population. Although it is an accepted method of incidence estimation, it means our estimate is based on a small number of cases, and some of the variation may be due to random error. In addition multiplier methods rely on many assumptions. In particular, we assumed that those who presented to the two tertiary referral hospital study sites were representative of the community sampled in the healthcare utilisation survey and that the care seeking behaviour of those surveyed is representative of the population. We are unaware of any substantial changes to the health system or health seeking behaviour from 2007 through 2014, and hence any healthcare utilisation survey error is likely to be consistent between the studies. In the 2007–08 study, Biggs et al identified differences between the age and sex distribution of the 2002 census population and those who participated in the healthcare utilisation survey [9]. We were unable to compare the demographics of the population in the 2012 census to those who took the healthcare utilisation survey but it is possible that the survey respondents are not representative. In order to understand the effect that changes in care-seeking behaviour would have on our estimate, we have performed a sensitivity analysis to provide a range of estimates. In this sensitivity analysis we varied the multipliers according to different questions from the healthcare utilization survey asking about fever shorter than 3 days. The larger multipliers derived by these questions reflect the fact that patients with shorter durations of fever are less likely to present to tertiary hospitals. We think that these estimates are less accurate than our final estimate as three quarters of our participants reported a fever of at least 3 days.

We are likely to have underestimated the incidence of disease through our use of serological diagnosis and enrolment from referral hospitals and may have biased inclusion towards those with severe disease. Firstly, we assumed that those who provided serum for testing were similar to those who did not. Secondly, we used test multipliers to attempt to account for the insensitivity of MAT in the acute phase of illness. We performed calculations using a sensitivity of 100% for MAT on paired serum samples and 48% for MAT on those providing only acute serum samples to ensure comparability with the 2007–08 incidence estimate. However, there is increasing evidence that MAT is less sensitive than the values of 100% for paired sera and 48.7% for solely acute serum that we used. It is therefore likely that the true incidence is towards the upper end of the estimate in our sensitivity analysis [16, 18, 19].

In conclusion, our study demonstrates substantial variation in leptospirosis incidence between time periods at the same site in continental Africa. Leptospirosis incidence appears to have declined from 2007–08 to 2012–14, although it remains an important cause of fever. This appears partly due to unstable transmission of a serovar from within the Mini serogroup. Our findings indicate the value of leptospirosis surveillance over multiple year time periods to understand the epidemiology of the disease. Our findings also highlight that much more work is needed to identify the animal reservoirs of *Leptospira* spp. in Africa in order to understand the human epidemiology.

### Disclaimer

The findings and conclusions in this report are those of the authors and do not necessarily represent the official position of the Centers for Disease Control and Prevention. Use of trade names and commercial sources is for identification only and does not imply endorsement by the US Department of Health and Human Services or the Centers for Disease Control and Prevention.

#### Presented in part

65th American Society of Tropical Medicine and Hygiene annual meeting, Atlanta, GA, 13–17 November 2016, abstract 1291

## Supporting Information

S1 Checklist STROBE checklist (DOC)
